# Deficiency of the Synaptic Adhesion Protein Leucine‐Rich Repeat Transmembrane Protein 4 Like 1 Affects Anxiety and Aggression in Zebrafish

**DOI:** 10.1111/apha.70042

**Published:** 2025-04-04

**Authors:** Eva Tatzl, Giulia Petracco, Isabella Faimann, Marco Balasso, Agnes Anna Mooslechner, Thomas Bärnthaler, Giovanny Rodriguez‐Blanco, Florian Reichmann

**Affiliations:** ^1^ Division of Pharmacology, Otto Loewi Research Center Medical University of Graz Graz Austria; ^2^ Clinical Institute of Medical and Chemical Laboratory Diagnostics Medical University of Graz Graz Austria; ^3^ BioTechMed‐Graz Graz Austria

**Keywords:** aggression, anxiety, leucine‐rich repeat transmembrane protein 4, neurotransmission, synaptic plasticity

## Abstract

**Aim:**

Leucine‐rich repeat transmembrane proteins (LRRTMs) are synaptic adhesion proteins that regulate synapse development and function. They interact transsynaptically with presynaptic binding partners to promote presynaptic differentiation. Polymorphisms of *LRRTM4*, one of the four members of this protein family, have been linked to multiple neuropsychiatric disorders and childhood aggression, but the underlying mechanisms and physiological function of LRRTM4 during behavior are currently unclear.

**Methods:**

To characterize the role of this gene for brain function, we combined a battery of behavioral assays with transcriptomic and metabolomic analyses, using zebrafish as a model system.

**Results:**

Our findings revealed that *lrrtm4l1*, a brain‐specific zebrafish orthologue of human *LRRTM4*, exhibits a brain region‐specific expression pattern similar to humans, with strong expression in the dorsal telencephalon, a brain area critical for regulating emotional‐affective and social behavior. *lrrtm4l1* ^−/−^ zebrafish displayed heightened anxiety and reduced aggression, while locomotion and social behavior remained unaffected by the gene knockout. Transcriptomic analysis of the telencephalon identified over 100 differentially expressed genes between wild‐type and mutant zebrafish and an enrichment of pathways related to synaptic plasticity and neuronal signaling. The brain metabolome of *lrrtm4l1* ^−/−^ zebrafish showed multiple alterations, particularly in the dopaminergic and adenosinergic neurotransmitter systems.

**Conclusion:**

These findings suggest that LRRTMs may have functions beyond their established role in excitatory synapse development, such as the regulation of neurotransmission and behavior. Targeting LRRTM4 therapeutically may thus be an interesting novel approach to alleviate excessive aggression or anxiety associated with a number of neuropsychiatric conditions.

## 1 Introduction

Leucine‐rich repeat transmembrane proteins (LRRTMs) are synaptic adhesion proteins regulating synapse development and function [1]. To date, four members of this protein family (LRRTM 1–4) have been identified in humans with different central nervous system expression patterns and functions. As the name suggests, they all contain leucine‐rich repeats in their extracellular domain enabling them to form contacts with presynaptic binding partners [2]. In contrast to LRRTM1‐3, LRRTM4 interacts transsynaptically preferentially with heparan sulfate proteoglycans (HSPGs) such as glypicans. This binding can induce presynaptic differentiation in the axons and plays a significant role in excitatory synapse development [3] In addition, via its cytoplasmic component, LRRTM4 regulates the location of glutamate receptor subunits and scaffolding proteins at the postsynaptic membrane [1]. Knockdown of *LRRTM4* in cultured cortical neurons as well as germ‐line knockout of *Lrrtm4* in mice have both revealed a significant decrease in dendritic spine density [4, 5]. In the dentate gyrus of the hippocampus, this finding is accompanied by reductions in excitatory synapse density and impaired activity‐regulated alpha‐amino‐3‐hydroxy‐5‐methyl‐4‐isoxazole propionic acid (AMPA) receptor trafficking [4]. Recently, it has been suggested that LRRTM4 is not only important for excitatory synapse development but also for the function of inhibitory synapses. In the retina, LRRTM4 is expressed by rod bipolar cells and found in GABAergic synapses, where it regulates the organization of synaptic ribbons [6]. In addition, sensory deprivation prevents adequate clustering of LRRTM4 at rod cell synapses, impairing the function of GABAergic synapses [7].

LRRTM4 has also caught the attention of the community because it has been linked to multiple neuropsychiatric disorders. Copy number variations near the *LRRTM4* locus, cell‐type specific LRRTM4 enrichment, as well as a duplication of the terminal *LRRTM4* exon have been linked to autistic traits, autism spectrum disorder, and Tourette syndrome [8, 9, 10]. Furthermore, *LRRTM4* polymorphisms have been associated with children's aggressive behavior, as well as attempted suicide in patients suffering from bipolar disorder [11, 12, 13]. However, how LRRTM4 disruption leads to behavioral abnormalities is currently unknown.

Zebrafish are an emerging model in biomedical research. This small cyprinid fish species displays a wide array of behaviors that can be reliably assessed in established behavioral assays. They are also readily amenable for genetic manipulation and feature unique properties such as larval transparency and high fertility, which enable, for example, in vivo whole brain imaging or high throughput drug screens [14, 15, 16]. The zebrafish genome is similar to the human genome, given that 71.4% of all human genes and 82% of disease‐related genes have at least one zebrafish orthologue [17]. In the current study we decided to study *lrrtm4l1*, one of the zebrafish orthologues of human *LRRTM4* that is highly expressed in the brain of embryonic and larval zebrafish, but not in other tissues [18]. Given the association of LRRTM4 polymorphisms with neuropsychiatric disease, we decided to investigate whether this gene regulates social and emotional‐affective behavior. In addition, to gain mechanistic insights, we analyzed the brain expression pattern of the gene in adult zebrafish and performed neurotranscriptomic and metabolomic analysis.

## 2 Results

### 2.1 Lrrtm4l1 Shows a Distinct Expression Pattern Throughout the Zebrafish Brain

**FIGURE 1:**
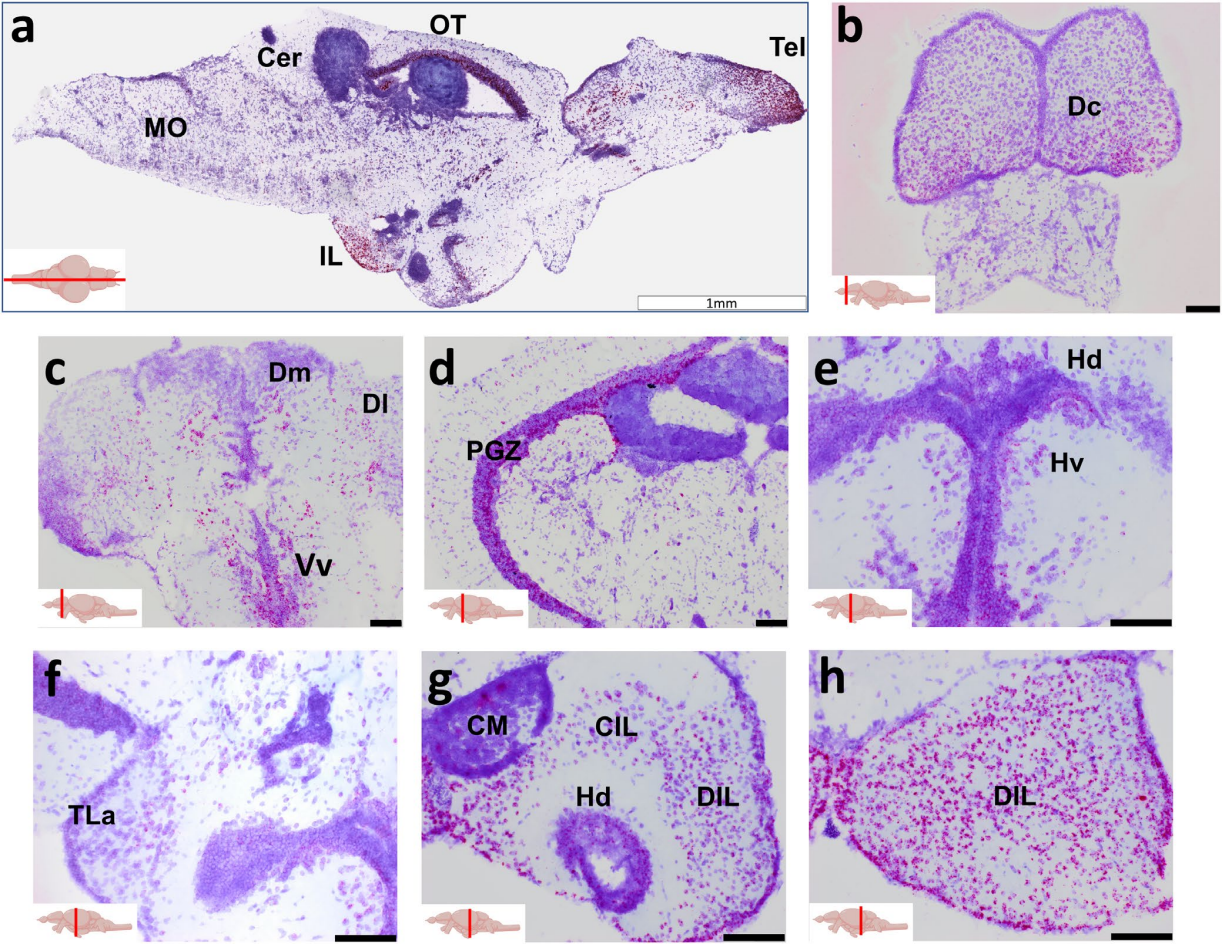
Lrrtm4l1 expression in the adult zebrafish brain. (a) Midsagittal section showing intense lrrtm4l1 expression (purple) in the telencephalon (Tel), inferior lobe (IL) and optic tectum (OT). (b–e) Coronal sections of the telencephalon (b, c), optic tectum (d), hypothalamus (e, f) and inferior lobe (g, h). Sections have been counterstained with thionine acetate for anatomical orientation. Scale bars are 1 mm in (a) and 100 μm in (b–h). The red line in the brain inserts indicates the sectioning plane. Cer, Cerebellum; CIL, central nucleus of the inferior lobe; CM, mamillary bodies; Dc, central zone of the dorsal telencephalon; DIL, diffuse nucleus of the inferior lobe; Dl, lateral zone of the dorsal telencephalon; Dm, Medial zone of the dorsal telencephalon; Hd, dorsal zone of the periventricular hypothalamus; Hv, ventral zone of the periventricular hypothalamus; IL, inferior lobe, MO, medulla oblongata; OT, optic tectum; PGZ, periventricular gray zone of optic tectum; Tel, telencephalon; TLa, torus lateralis; Vv, ventral zone of the ventral telencephalon. Schematic zebrafish brain inserts created by BioRender.com.

Expression studies in mammals found a wide distribution of *LRRTM4* transcripts in the human and mouse brain, with particularly high expression levels in the amygdala and hippocampus [2], two brain areas that play an important role in emotional–affective behavior, learning, and memory. To investigate whether this expression pattern is similar in the zebrafish brain, we used in situ hybridization to study the expression of *lrrtm4l1*, one of the zebrafish orthologues of *LRRTM4*. Analysis revealed a brain region‐specific *lrrtm4l1* expression pattern with particularly high expression levels in the anterior telencephalon (Tel), the inferior lobe (IL) and the optic tectum (OT), but only low to no expression in many other brain areas such as the cerebellum (Cer) and medulla (MO) (Figure 1a). In the telencephalon, multiple areas showed moderate to strong expression levels, including the central zone of the dorsal telencephalon (Dc, Figure 1b), the ventral zone of the ventral telencephalon (Vv), but also the medial zone (Dm) and the lateral zone of the dorsal telencephalon (Dl) (Figure 1c), the zebrafish homologues of the mammalian amygdala and hippocampus, respectively [19]. In the optic tectum, strong *lrrtm4l1* expression levels were observed in the periventricular gray zone (PGZ), while other parts of the OT showed only low expression levels (Figure 1d). Weak to moderate staining was also observed in the hypothalamus, particularly in the ventral zone of the periventricular hypothalamus (Hv), the dorsal zone of the periventricular hypothalamus (Hd) (Figure 1e) and the lateral torus (TLa) (Figure 1f). In the inferior lobe, we found intense staining in the diffuse nucleus of the inferior lobe (DIL) and the central nucleus of the inferior lobe (CIL), moderate staining in the (Hd), but no staining in the directly adjacent mamillary bodies (CM) (Figure 1g,h).

### 2.2 Lrrtm4l1 Knockout Increases Anxiety

**FIGURE 2:**
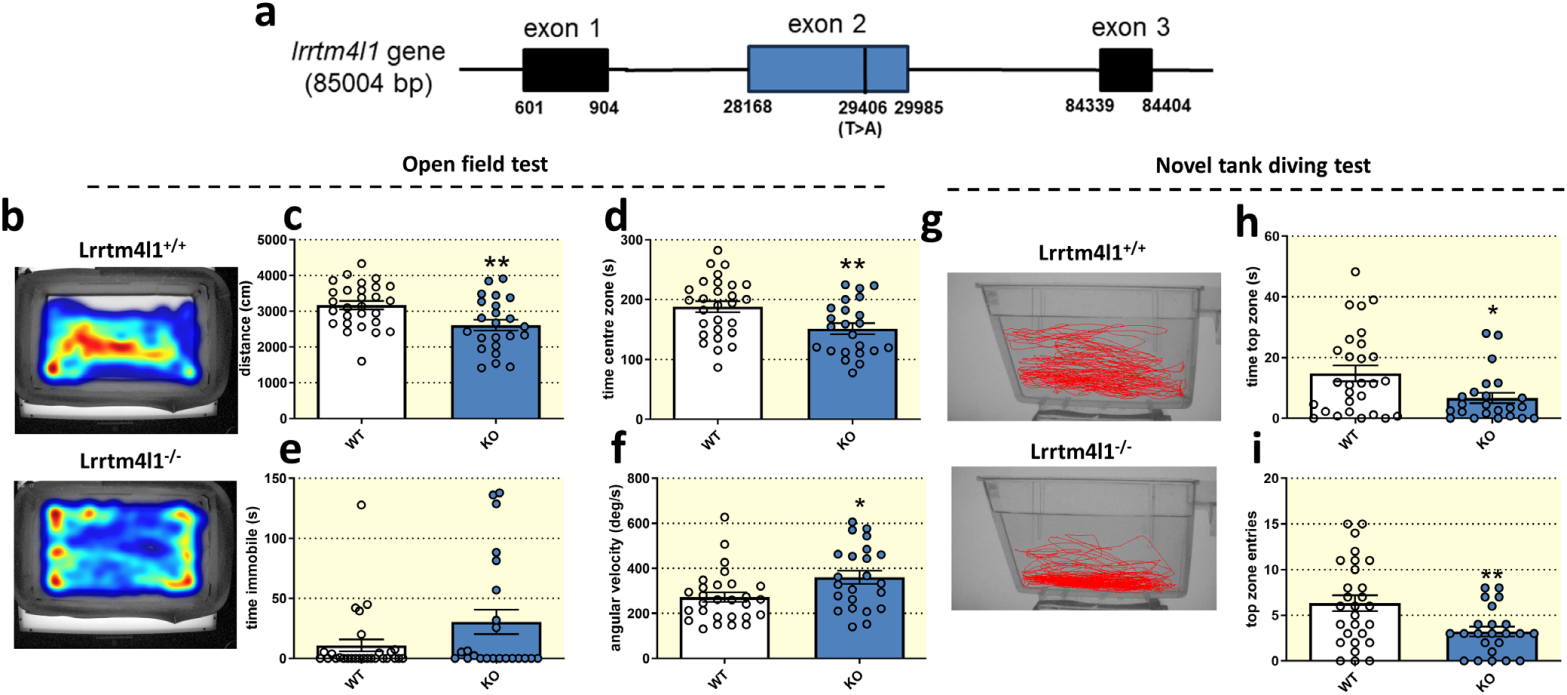
lrrtm4l1^−/−^ zebrafish display heightened anxiety. (a) Schematic representation of the lrrtm4l1 gene. The black line within exon 2 indicates the position of the nonsense mutation of the sa21708 zebrafish mutant line. Numbers represent bp positions. (b) Representative tracking heatmaps of lrrtm4l1^+/+^ (WT) and lrrtm4l1^−/−^ (KO) zebrafish in the open field test. (c) KO zebrafish swim a shorter distance and (d) spend less time in the centre zone of the open field test. (e) KO animals show no difference in time spent immobile, but (f) display increased angular velocity. (g) Representative swimming trajectories of WT and KO zebrafish in the novel tank diving test. (h) KO zebrafish spend less time in the top zone and (i) enter the top zone of the test tank less frequently. *n* = 23‐28/group. Student's *t* test or Mann–Whitney *U* test. **p < 0.01;*p < 0.05 KO versus WT. Data are presented as mean ± SEM.

Given this localized *lrrtm4l1* expression pattern in brain areas important for emotional‐affective and social behavior, we next investigated the role of this gene for multiple behavioral domains. For this, we made use of a zebrafish mutant line (sa21708) that is characterized by a nonsense mutation (T>A) in exon 2 of the *lrrtm4l1* gene leading to a premature stop codon (Figure 2a). The mutation affects both *lrrtm4l1* transcripts resulting in considerably truncated proteins and preventing the translation of the transmembrane and the cytosolic domain (Figure S1). Homozygous mutant fish (lrrtm4l1^−/−^) carrying this allele develop normally and do not show obvious phenotypical changes compared to corresponding wild‐type fish (WT, lrrtm4l1^+/+^), as suggested by a similar macroscopic appearance, size, and weight (Figure S2). However, when analyzing behavior, lrrtm4l1^−/−^ zebrafish showed strong alterations compared to lrrtm4l1^+/+^ fish. In the open field test (OFT), an assay used to study general locomotion and anxiety, we found that adult mixed‐sex lrrtm4l1^−/−^ fish moved less than corresponding WT fish (Figure 2b). In addition, they spent less time in the center zone of the test tank (Figure 2c) indicating heightened anxiety levels. Although the time spent immobile during the assay was only nominally increased (Figure 2d), the mutant fish showed higher angular velocity values (Figure 2e), a readout of erratic swimming, also suggesting enhanced anxiety. To further investigate the effects of the mutation on anxiety, we performed the novel tank diving test (NTD). Like in the OFT, lrrtm4l1^−/−^ zebrafish were more anxious than corresponding WT fish. Specifically, lrrtm4l1^−/−^ individuals spent less time in the top zone of the tank (Figure 2f) and entered the top zone less frequently (Figure 2g), both suggesting enhanced anxiety. As a third test to evaluate anxiety in the mutant fish, we used the light/dark preference test. Although the time spent in the light compartment was not changed between the two genotypes, mutants entered the light compartment less frequently (Figure S3) again indicating enhanced anxiety.

### 2.3 Lrrtm4l1 Knockout Reduces Aggression, but Does Not Alter Social Behavior

**FIGURE 3:**
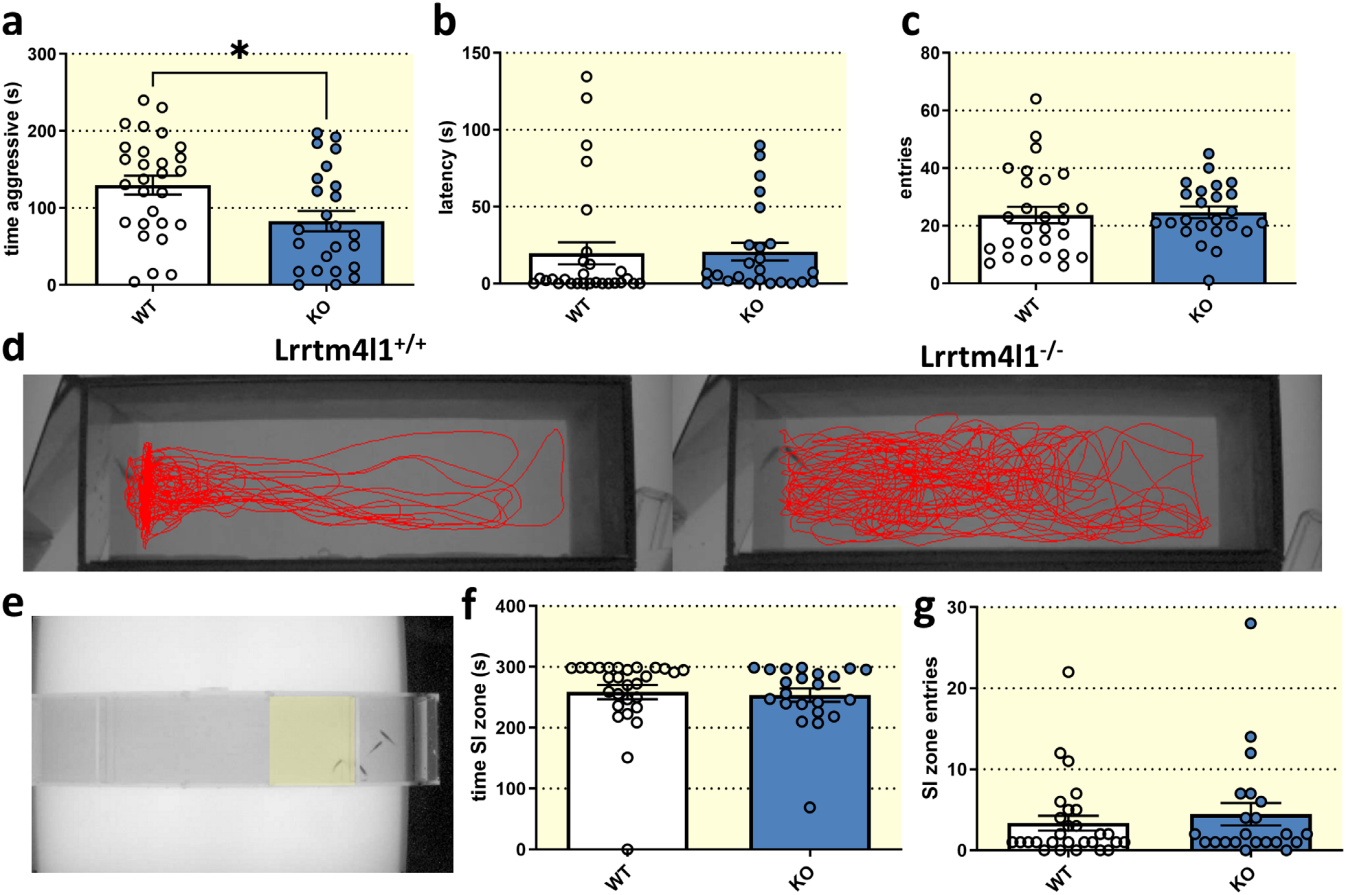
lrrtm4l1^−/−^ zebrafish are less aggressive. (a) Lrrtm4l1^−/−^ (KO) zebrafish were less aggressive than lrrtm4l1^+/+^ (WT) zebrafish during the mirror‐induced aggression (MIA) assay. (b) The genotype did not alter the latency to approach the mirror and (c) entries into the mirror zone. (d) Representative swimming trajectories of WT and KO zebrafish in the MIA test. (e) Test setup of the corridor social interaction (SI) test showing a test fish approaching the stimulus shoal. The social interaction zone is virtually highlighted in yellow. (f) WT and KO zebrafish spend an equal amount of time in the SI zone and (g) enter the SI zone equally often. *n* = 22‐28/group. Student's *t* test or Mann–Whitney *U* test. *p < 0.05 KO versus WT. Data are presented as mean ± SEM.

To analyze the effects of *lrrtm4l1* knockout on other behavioral domains, we next assessed the aggressive behavior of the adult mutant fish using the mirror‐induced aggression assay (Figure 3a–d [20]). Interestingly, we found that *lrrtm4l1* mutant zebrafish were less aggressive against their own mirror image compared to WT fish (Figure 3a). We did not detect a difference in the latency to approach the mirror (Figure 3b) and also saw no change in the number of mirror approaches (Figure 3c) suggesting that the reduced aggressiveness is not related to enhanced anxiety during the assay, but might rather be a consequence of altered perception of the opponent and/or reduced willingness to attack. To assess whether the reduced aggressiveness is a result of abnormal general social behavior, we performed the corridor social interaction test [21]. In this assay, a test fish explores a corridor‐like tank and has the choice between staying in the vicinity of a stimulus fish group or avoiding social interactions at the other end of the tank (Figure 3e). Analysis revealed that both lines had an equal preference for the social interaction zone of the tank as measured by the time spent near the stimulus fish (Figure 3f) and also the entries into the social interaction zone (Figure 3g). We also detected no genotype difference in the social novelty assay, where test fish are given the choice to interact with a known or unknown shoal, suggesting that general social behavior is not affected by *lrrtm4l1* deletion (Figure S4).

### 2.4 *Lrrtm4l1* Knockout Alters Genes and Pathways Related to Synaptic Plasticity, Neural Connectivity, and Lipid Metabolism

**FIGURE 4:**
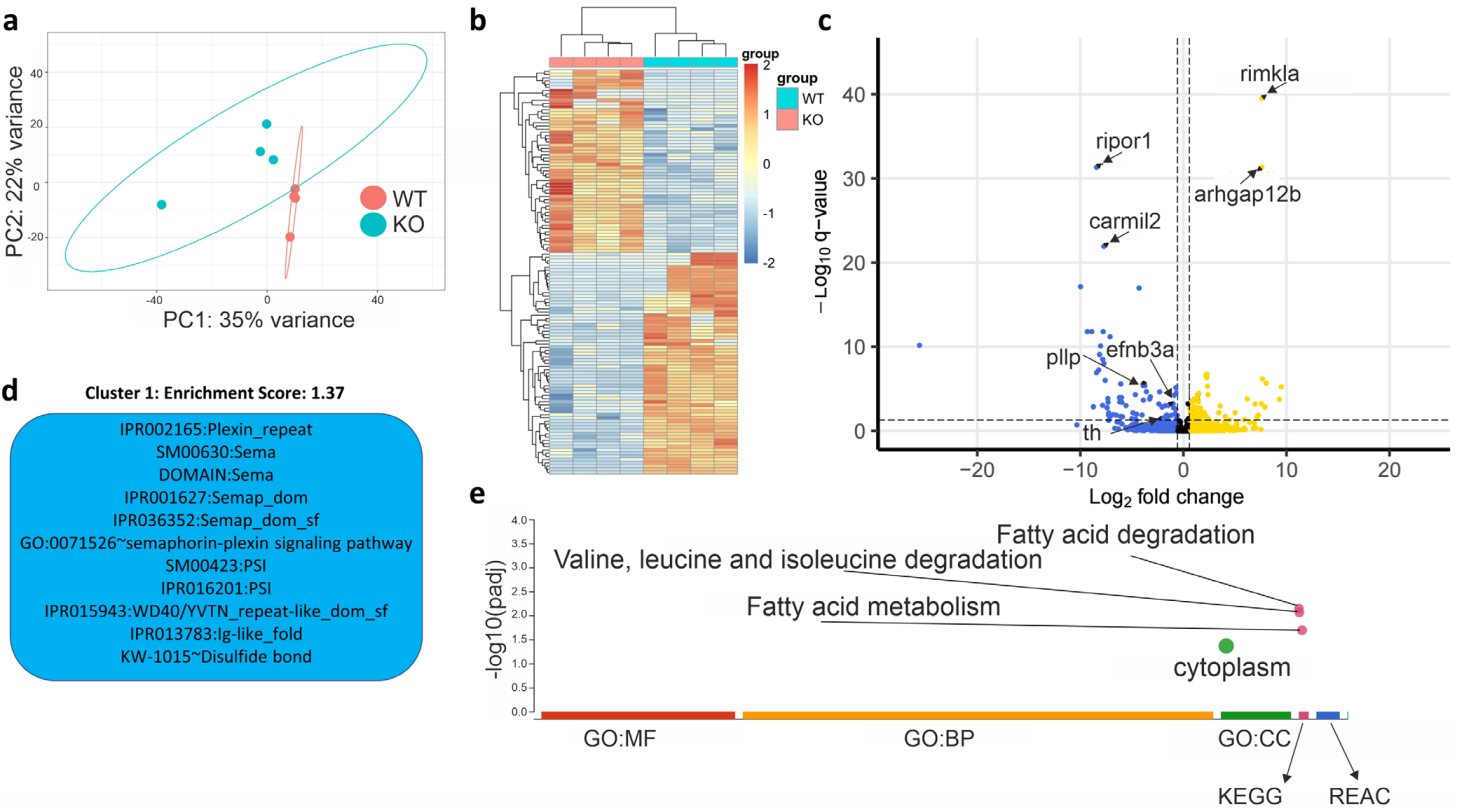
Neurotranscriptomic effects of lrrtm4l1 deletion. (a) Principal component analysis plot of the 200 most variable genes after differential expression analysis between lrrtm4l1^−/−^ (KO) and lrrtm4l1^+/+^ (WT) zebrafish. (b) Heatmap of differentially expressed genes (DEGs; *p*adj < 0.05 and LFC > |0.58|) between KO and WT zebrafish. Hierarchical clustering of samples and genes reveals large differences between KO and WT, but similar transcriptional patterns within the two lines. (c) Volcano plot displaying the DEGs between KO and WT. Selected genes have been highlighted. Golden dots indicate genes upregulated in KO more than log fold change 0.58, blue dots represent genes downregulated in HAZ more than LFC—0.58 and black dots represent genes not passing these thresholds as indicated by vertical dotted lines. The horizontal dotted line indicates the significance threshold (*p*adj 0.05) (d) Functional annotation clustering using DAVID pathway analysis revealed a significantly enriched cluster (enrichment score ≥ 1.3) related to semaphoring‐plexin signaling. (e) g:Profiler functional enrichment analysis revealed enriched KEGG pathways related to fatty acids and amino acids. *n* = 4/group.

To study the molecular consequences of *lrrtm4l1* knockout, we analyzed differences in gene expression between lrrtm4l1^−/−^ and lrrtm4l1^+/+^ fish by RNAseq. For this, we focused on the telencephalon, because this brain area showed strong *lrrtm4l1* expression in this study and it contains important brain areas linked to anxiety and aggression [22]. Principal component analysis of the dataset revealed a clear separation of mutant and WT samples into 2 distinct clusters, indicating a significant change in the brain transcriptome of the mutant zebrafish (Figure 4a). In line with this finding, differential expression analysis revealed 126 differentially expressed genes (DEGs; *p*adj < 0.05 and LFC > |0.58|) between the 2 genotypes, with 57 DEGs upregulated in lrrtm4l1^−/−^ zebrafish and 69 DEGs downregulated (Figure 4b; Table S1). Hierarchical clustering of samples revealed a clear separation between lrrtm4l1^−/−^ and lrrtm4l1^+/+^ zebrafish indicating similar transcriptional patterns within the two genotypes (Figure 4b). The most significant DEGs are *ribosomal modification protein rimK‐like family member A* (*rimkla*) and *rho GTPase activating protein 12b (arhgap12b)* that are upregulated in mutant fish and *RHO family interacting cell polarization regulator 1 (ripor1)* and *capping protein regulator and myosin 1 linker 2 (carmil2)* that are downregulated (Figure 4c.) Among those, *rimkla* and *arhgap12b* are of particular interest, as they have been linked to behavior and synaptic function, respectively [23, 24]. Other interesting DEGs that were all downregulated in the mutant zebrafish include *tyrosine hydroxylase (th)*, the rate limiting enzyme in the biosynthesis of catecholamines [25], *plasmolipin (pllp)*, a main component of myelin sheaths [26] and *ephrin‐B3a (efnb3a)*, which has also been linked to synaptic plasticity [27] (Figure 4c). Pathway analysis using DAVID software [28] revealed a significantly enriched cluster (enrichment score 1.37) mainly containing terms involved in semaphorin‐plexin signaling (Figure 4d), a biological process important for the homeostasis and morphogenesis of many tissues and recognized for its role in axon guidance and neural connectivity [29]. Specifically, we found that *sema domain, immunoglobulin domain (Ig), short basic domain, secreted, (semaphorin) 3B* (*sema3b*) and *sema domain, immunoglobulin domain (Ig), short basic domain, secreted, (semaphorin) 3bl* (*sema3bl*) were upregulated in the mutant zebrafish, while the semaphorin receptor *plexin b2b* (*plxnb2b*) was downregulated (Figure S5) In addition, g:profiler functional enrichment analysis [30] revealed an overrepresentation of the KEGG pathway terms fatty acid metabolism, fatty acid degradation as well as valine, leucine and isoleucine degradation indicating altered lipid and amino acid metabolism in the mutants (Figure 4e). The differentially expressed genes contributing to this result comprise *acyl‐CoA dehydrogenase short/branched chain* (*acadsb*) and *trans‐2,3‐enoyl‐CoA reductase a* (*tecra*) which are downregulated in *lrrtm4l1* ^−/−^ zebrafish as well as *acyl‐CoA dehydrogenase, short/branched chain‐like* (*LOC100037342*) and *aldehyde dehydrogenase 3 family member A2* (*aldh3a1*), which are upregulated (Figure S6). While the function of acadsb, aldh3a1 and LOC100037342 in the CNS are currently unknown, it has been found that mutations of *TECR*, the human orthologue of zebrafish *tecra*, can cause autosomal recessive nonsyndromic mental retardation [31]. The gene encodes a fatty acid synthetase that is involved in the synthesis of very long‐chain fatty acids. Interestingly, it also shows high expression in cerebrovascular endothelial cells and is important for the maintenance of the blood–brain barrier [32].

### 2.5 *Lrrtm4l1* Knockout Modulates Dopaminergic and Adenosinergic Neurotransmitter Signaling

**FIGURE 5:**
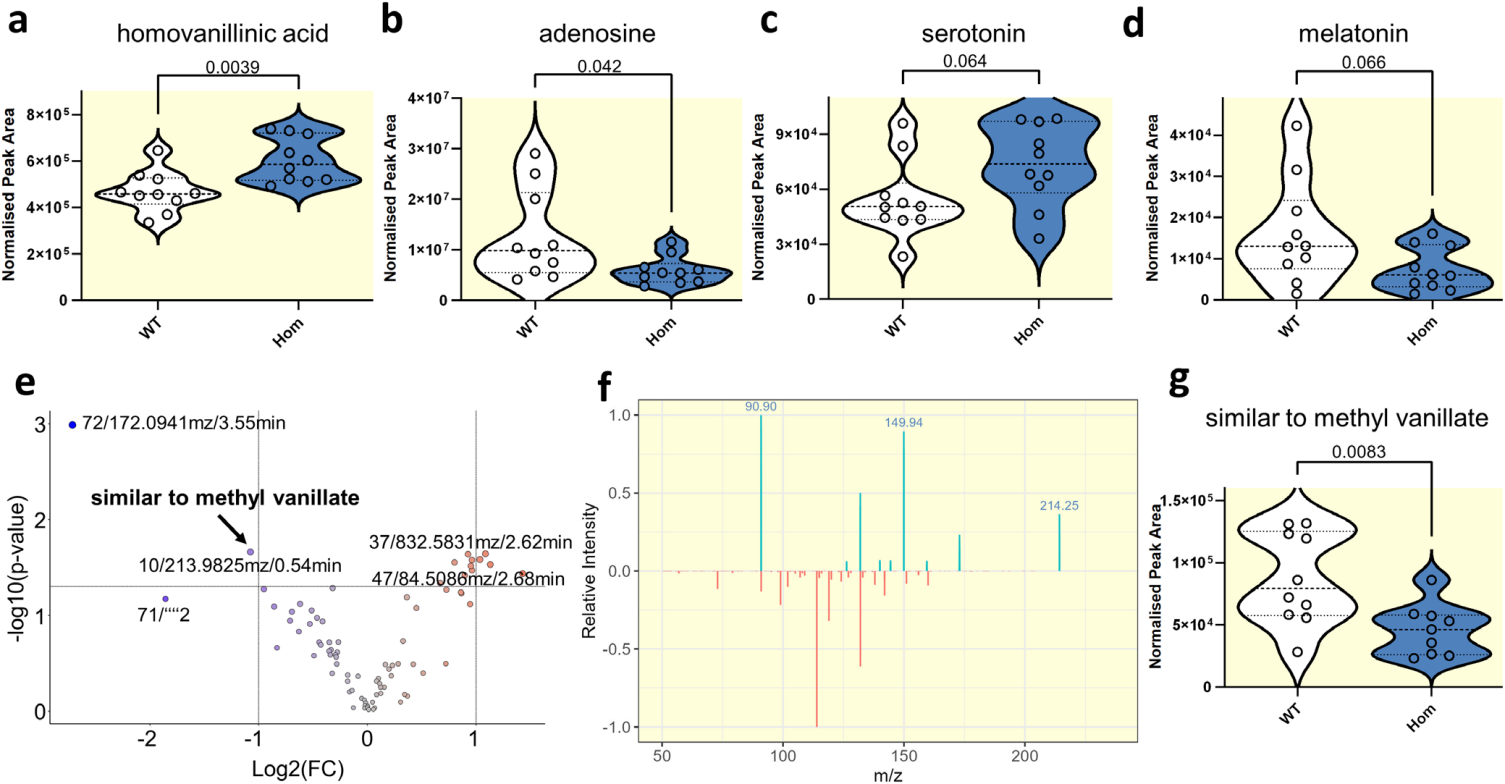
Neurotransmitter and brain metabolome changes in *lrrtm4l1* ^*−/−*^ zebrafish. Telencephalic levels of (a) homovanillinic acid, (b) adenosine, (c) serotonin and (d) melatonin as measured by targeted metabolomics in *lrrtm4l1* ^*−/−*^ (Hom) and lrrtm4l1^+/+^ (WT) zebrafish. *n* = 10/group. Student's *t* test. Data are presented as violin plots with the horizontal black line indicating the median. (e) Volcano plot displaying metabolites detected by untargeted metabolomics. Metabolites with adjusted *p*‐values below 0.05 and a log2fold change ≥ |1| were regarded as significant. (f) Spectral matching of detected metabolites. (g) Violin plot displaying telencephalic levels of a metabolite similar to methyl vanillate. *n* = 9–10/group.

To study if *lrrtm4l1* deletion affects the brain metabolome, we used mass spectrometry‐based metabolomics. Again, focusing on the telencephalon, we performed targeted metabolomics of neurotransmitters and related metabolites. We were able to detect 19 different molecules in the zebrafish brain tissue (Figure 5; Figure S7). Among them, homovanillic acid (HVA) levels were significantly higher in *lrrtm4l1* ^−/−^ zebrafish (Figure 5a), while adenosine levels were significantly lower (Figure 5b) suggesting alterations in the dopaminergic and adenosinergic neurotransmitter systems, respectively. In addition, we found a trend toward higher serotonin (*p* = 0.064; Figure 5c) and lower melatonin levels (*p* = 0.066; Figure 5d) in the mutants. We also performed unbiased metabolomics analysis on the same extracted tissue samples. We found a total of 1555 metabolite features, and after correcting by FDR, only 6 metabolite features were de‐regulated (Figure 5e). Two of those features at m/z 71 and 84 could be in‐source fragments, which are difficult to annotate. We used the publicly available GNPS platform to annotate the metabolite features when MS2 information was available. We found two up‐regulated features with m/z values of 832.5831 and 732.5524, with fragmentation profiles similar to phospholipids. The metabolite feature with m/z 172.0941 was the most down‐regulated feature, but it was not possible to annotate with the spectral libraries matched. Interestingly, although most of these metabolite features cannot be unequivocally annotated, we found a metabolite feature with a m/z of 213.9825 that resulted in a spectral similarity to methyl vanillate (mass diff of 30.76 Da), a derivative of vanillic acid (Figure 5f,g).

## 3 Discussion

Synaptic adhesion proteins, including LRRTM4, are important regulators of synaptic transmission and therefore also of brain function. Human genome‐wide association studies have linked LRRTM4 polymorphisms to a variety of neuropsychiatric disorders [8, 9, 10, 11, 13] and also to childhood aggression [12], but the underlying mechanisms are largely unknown. In the current work, we used zebrafish to study if *lrrtm4l1*, the zebrafish orthologue of *LRRTM4*, influences behavior, neurotranscriptomics, and the brain metabolome. We found that *lrrtm4l1* shows a brain region‐dependent expression pattern with strong expression in telencephalic brain areas important for behavior. Deletion of *lrrtm4l1* increases anxiety and reduces aggression of mutant zebrafish, a phenotype accompanied by multiple changes in gene expression and metabolites within this brain area. Most notably, we detected dysregulations of semaphorin‐plexin signaling, dopaminergic and adenosinergic neurotransmission, and lipid acid metabolism.

As a first step to analyze the role of *lrrtm4l1* in the zebrafish brain, we investigated its gene expression pattern. In line with studies in mammals [2], we found regional differences in gene expression levels across the brain. Many brain areas such as the cerebellum or medulla, but also large parts of the mes‐ and diencephalon showed very low expression levels. In contrast, the gene is highly expressed in the telencephalon. This is, according to the Allen human brain atlas and the genotype‐tissue expression (GTEx) portal [33], similar to the human *LRRTM4* brain expression pattern with high expression levels in cortical areas, the hippocampal dentate gyrus, amygdala, and hypothalamus, but low expression in the cerebellum. Of particular note is the high expression level in the Dm, which is considered the zebrafish orthologue of the mammalian amygdala [22]. This is consistent with *LRRTM4* expression in the human brain, which is also highly expressed in the amygdala [2]. Given that the amygdala is an essential brain area for processing fear and emotions [34], it seems likely that the observed anxiety phenotype of lrrtm4l1^−/−^ zebrafish is a result of disturbed lrrtm4l1 function within this brain area. Another telencephalic brain area that showed high *lrrtm4l1* expression is the Dl, which is considered the homologue of the mammalian hippocampus [35]. This is the brain structure that has been most investigated in mammalian LRRTM4 studies. In the human brain, LRRTM4 is highly expressed in the granular cell layer of the dentate gyrus, a subregion of the hippocampus, and it has been found to bind to a different presynaptic binding partner than other LRRTMs, namely heparan sulfate proteoglycans (HSPGs). It has also been suggested that LRRTM4 regulates excitatory but not inhibitory synapse development within this area [3]. Dl and Dm are regarded as parts of the zebrafish social decision‐making network (SDMN) [15]. Across species, this network of brain areas is essential for the regulation of multiple forms of social behavior [36]. In zebrafish, it has been shown that many nodes of the SDMN are activated after aggression [37]. In addition, it is known that aggression‐induced neuronal activation of the SDMN depends on genetic factors. For example, we have previously shown that *histamine 3 receptor* (*hrh3*) knockout zebrafish that are characterized by reduced mirror aggression show changes in basal and aggression‐induced neuronal activity in the SDMN, particularly in the Dm and the Vv [38]. Given that *lrrtm4l1* knockout fish are also less aggressive than WT fish and that *lrrtm4l1* is strongly expressed in brain areas of the SDMN including Dm, Dl, and Vv, it might be possible that the reduced aggressiveness of *lrrtm4l1* mutants is linked to changes of neuronal activity especially in these brain areas.

Interestingly, we also found very strong *lrrtm4l1* expression in the inferior lobe, a brain area forming a macroscopic bump on the ventral side of the zebrafish brain. It has been suggested based on connectivity data that this area is essential for multimodal sensory integration [39]. In zebrafish larvae, the area is activated by presenting moving objects on a screen to the fish, suggesting a role in visual information processing [40]. In line with this finding, we also detected strong expression in the optic tectum, homologous to the superior colliculus in mammals, which is essential for detecting and processing sensory stimuli [41]. The strong expression of *lrrtm4l1* in both of these structures indicates a potential role of this gene for visual processes. Interestingly, as mentioned above, a link between LRRTM4 and vision has already been established in a previous study [6]. Specifically, LRRTM4 has been found at GABAergic synapses on axon terminals of mouse rod bipolar cells regulating synapse function and arrangement of synaptic ribbons. Additionally, dark‐rearing of mice prevents maturation of GABAergic synapses on axons of bipolar cells, an effect associated with reduced expression levels of LRRTM4 [7]. The behavioral analysis in this study did not point to obvious visual impairments of *lrrtm4l1* knockout animals, but further studies are needed to investigate this systematically, for example, by specific behavioral testing or analysis of *lrrtm4l1* function in the zebrafish retina.

Multiple recent genome‐wide association studies (GWAS) have found a link between LRRTM4 and neuropsychiatric disease [8, 9, 10, 13, 42, 43], but the mechanisms underlying these findings are poorly understood. Here, we report for the first time to our knowledge on the emotional‐affective and social behavior of animals with a null mutation of a human *LRRTM4* orthologue. Our data suggest that the gene regulates anxiety‐like behavior, given that *lrrtm4l1* knockout zebrafish are more anxious in multiple behavioral assays. This is in line with the claustrophobia‐like phenotype of *Lrrtm1* knockout mice, another member of the LRRTM protein family, that are known to avoid small enclosures [44]. In a translational view, the findings of enhanced anxiety fit well with reported comorbidities of diseases that have already been linked to LRRTM4, including ASD and Tourette [9, 10]. In fact, anxiety disorders are one of the most common psychiatric comorbidities of autism spectrum disorder patients, which impair the quality of life of affected individuals and increase the possibility of worse long‐term outcomes [45]. Similarly, it has been shown that patients with Tourette syndrome have high rates of comorbid anxiety and depression [46]. *LRRTM4* has not come up as a significant hit in GWAS investigating anxiety disorders directly so far, which may be related to the large heterogeneity of anxiety disorders raising the question of whether specific subforms of anxiety disorders are associated with *LRRTM4* dysfunction. It will also be interesting to investigate in future studies whether ASD or Tourette patients with and without anxiety disorders show differences in the genetic makeup of the *LRRTM4* locus. The second major behavioral alteration detected in this study is reduced aggression. Although conceptually this phenotype might be the consequence of enhanced anxiety, our analysis points to an independent phenotype, given that *lrrtm4l1* ^−/−^ zebrafish did not show typical signs of anxiety during the aggression assay. Reduced aggression of the mutant animals is moreover in line with a human GWAS that noted an association of multiple SNPs around the *LRRTM4* locus with children's aggressive behavior [12]. This meta‐analysis of 9 population‐based studies found that in a sample of over 18 000 children, the strongest signal emerged on chromosome 2 near the *LRRTM4* locus, a SNP that is related to an allele that promotes reduced aggression [12]. A link between LRRTM4 and aggression has also been found in a GWAS aiming to identify candidate genes underlying various phenotypic traits in dogs, including herding, predation, temperament, and trainability. *LRRTM4* was found to be one of the candidate genes governing predation behavior [47].

To investigate how *lrrtm4l1* deletion leads to behavioral alterations, we performed a detailed analysis of the lrrtm4l1^−/−^ telencephalon. At the transcriptional level, the most significant differences compared to WT animals were related to genes involved in neuronal signaling. Among those, *rimkla* is of particular interest, as it codes for the enzyme N‐acetylaspartylglutamate (NAAG) synthase II that synthesizes NAAG, a neuropeptide implicated in cognition and memory [24]. We found the gene to be upregulated in the mutant animals, suggesting that increased amounts of NAAG are synthesized. This can lead to a disturbance of the glutamatergic neurotransmitter system, given that glutamate is a substrate for NAAG synthase II and also a metabolite after NAAG degradation by glutamate carboxypeptidase II [24]. Furthermore, NAAG selectively binds to presynaptic metabotropic glutamate receptor type 3, inhibiting the release of glutamate, but also of the major neurotransmitters GABA and glycine [48]. Additionally, ARHGAP12, the mammalian orthologue of *arhgap12b*, which was upregulated in lrrtm4l1^−/−^ zebrafish, has been found to regulate excitatory synaptic structure and function [23]. Notably, ARHGAP12 promotes postsynaptic glutamate ionotropic AMPA receptor endocytosis [23], which is the opposite effect of LRRTM4 that is known to facilitate activity‐regulated clustering of AMPA receptors at the postsynaptic membrane [4]. It is currently unknown whether there is a direct interaction between these two synaptic signaling pathways, but the upregulation of *arghap12b* in our model suggests that the dysfunction of the transsynaptic regulator lrrtm4l1 also disrupts the activity of other synaptic proteins. In line with this idea, pathway analysis revealed semaphorin–plexin signaling alterations in the mutants. Semaphorins and plexins are well‐known regulators of axon guidance, neurogenesis, gliogenesis, and neural migration [49]. Semaphorin3b and plexin receptor b2, the orthologues of the differentially expressed semaphorin –lexin pathway genes in the *lrrtm4l1* mutants, have been implicated in axonal pathfinding and synapse formation, respectively [50, 51].

Another interesting finding of this study is the disruption of neurotransmitter signaling in *lrrtm4l1* mutants. Besides the potential glutamatergic dysregulation via NAAG and altered AMPA receptor trafficking as discussed above, we found that *th*, the rate‐limiting enzyme of catecholamine synthesis which catalyzes the conversion of L‐tyrosine to L‐3,4‐dihydroxyphenylalanine (L‐DOPA), is downregulated in *lrrtm4l1* ^−/−^ zebrafish. Although we did not detect changes in L‐DOPA or dopamine by targeted metabolomics, we found that homovanillic acid (HVA) levels are higher in the mutants. HVA is a major metabolite of dopamine degradation and the resulting increased ratio between HVA and dopamine in *lrrtm4l1* ^−/−^ zebrafish suggests increased dopamine turnover. This increased turnover of dopamine may have contributed to the behavioral phenotype of *lrrtm4l1* ^−/−^ zebrafish, considering that, for example, higher striatal dopamine turnover has been found in rats exposed to aggressive encounters [52]. Altered dopamine turnover rates have also been found in the context of anxiety, for instance, as one of the mechanisms mediating the anxiolytic effects of probiotics [53]. The function of LRRTM4 in dopaminergic synapses is currently unknown, but the observed alterations in the dopaminergic system after *lrrtm4l1* deletion and the expression of *lrrtm4l1* in brain areas receiving substantial dopaminergic synaptic input such as the ventral telencephalon [54] point to a possible role of LRRTM4 in dopaminergic neurotransmission. Noteworthy is also the significant decrease of adenosine in the mutant zebrafish telencephalon. Adenosine has numerous functions in the CNS including the regulation of the sleep–wake cycle, neuronal activity, and neurotransmitter release [55]. The adenosinergic system has also been implicated in anxiety disorders, given that caffeine and other adenosine receptor antagonists have anxiogenic properties in mammals [56]. In line with these findings, caffeine as well as selective adenosine A_1_ receptor antagonists have been shown to increase anxiety in zebrafish [57], which fits with the reduced adenosine concentration detected in the brains of the anxious *lrrtm4l1* mutants in this study. Adenosine has also been implicated in the aggressive behavior of mice. Specifically, adenosine analogues are able to counteract isolation‐induced aggressiveness [58] and mice lacking the adenosine A1 receptor show increased aggressiveness in the resident‐intruder task [59]. Thus, the altered telencephalic adenosine levels in this study might have contributed to the behavioral phenotype of the mutant animals, although the link between LRRTM4 and adenosine requires further investigation.

Finally, unbiased metabolomics analyses revealed reduced levels of a metabolite similar to methyl vanillate (m/z 213.9825) in the telencephalon of mutant zebrafish. This is interesting given that vanillin derivatives have been linked to neuroprotective effects [60] and the described anti‐oxidative properties of methyl vanillate [61]. However, further method development with authentic chemical standards along the methyl vanillate pathway or complementary techniques such as mass spectrometry imaging is required to better understand its link to the LRRTM proteins. In addition, we found two significant metabolic features with spectral similarity to phospholipids. Taking into account the RNA expression data, which also suggest fatty acid and lipid metabolism de‐regulation in the samples under study, further measurements using more hydrophobic settings in a lipidomics method could be performed in the future to complement the data generated in this work.

A limitation of this work is that it is unclear how our findings in zebrafish can be translated to humans. Although the zebrafish genome has around 70% homology to the human genome [17], a genome duplication event during evolution in teleost fish led to the duplication of many genes. This is also the case for the *LRRTM4* gene, where another orthologue, *lrrtm4l2*, exists in zebrafish. For future studies, it will be interesting to investigate how *lrrtm4l2* knockout affects zebrafish behavior and whether a double knockout of both orthologues leads to a stronger phenotype. It would also be interesting to study the effects of *Lrrtm4* knockout on behavior in other animal species such as mice or rats or to develop pharmacological tools targeting LRRTM4, which would enable the community to further evaluate the therapeutic potential of targeting the protein. Another potential line for further research is the effect of *lrrtm4l1* knockout on developmental or age‐related behavioral phenotypes. In this study, we focused on adult zebrafish and therefore cannot draw conclusions for other life stages. Finally, we cannot rule out the possibility that *lrrtm4l1* knockout leads to sex‐specific effects at the behavioral, transcriptomic, or metabolomic level because we did not perform sex‐specific analysis. Although we used mixed‐sex zebrafish, adequately powered additional studies would be needed to answer this research question. To our knowledge, there is currently no published evidence for sex‐specific *LRRTM4* alterations in any species.

## 4 Conclusions

In summary, the current paper investigated for the first time whether an orthologue of human *LRRTM4* regulates emotional‐affective and social behavior of animals. We showed that *lrrtm4l1* ^*−/−*^ zebrafish display increased anxiety and reduced aggression, suggesting an important function of the gene for these behavioral phenotypes. Transcriptomic and metabolomic analysis of the zebrafish telencephalon, which shows particularly high expression of *lrrtm4l1* in wild‐type animals, revealed alterations in synaptic plasticity and changes in neurotransmitter signaling in *lrrtm4l1* ^−/−^ zebrafish. These findings indicate additional LRRTM4 functions besides its role during excitatory synapse development, such as the regulation of neurotransmission in dopaminergic and adenosinergic synapses. In a translational view, it would be interesting to further characterize the potential of LRRTM4 as a treatment target to ameliorate excessive aggression or anxiety associated with a number of neuropsychiatric conditions.

## 5 Materials and Methods

### 5.1 Experimental Animals and Housing Conditions

Experiments were performed with mixed‐sex, age‐matched adult (6–12 months old) *lrrtm4l1* ^−/−^ and corresponding wild‐type (*lrrtm4l1* ^+/+^) zebrafish obtained from the local breeding colony maintained at the Medical University of Graz. *Lrrtm4l1* ^+/−^ zebrafish embryos (allele name: sa21708) generated by the zebrafish mutation project at the Wellcome Sanger Institute were obtained from the European zebrafish resource center (EZRC) at Karlsruhe Institute of Technology (KIT), raised, and incrossed to obtain homozygous mutants and corresponding WT fish. Genotyping was performed using rhAMPs assays (CD.GT.JSGT2882.1) from IDT (Leuven, Belgium). Animals used for experiments were obtained from multiple crosses and tanks to avoid batch effects. Mixed zebrafish groups were used to avoid male animal bias. All animals were kept at the same stocking density of 5–6 adult/fish per liter to ensure consistent environmental conditions. The zebrafish facility features optimal housing conditions for zebrafish (Tecniplast), a 14 h light/10 h dark cycle, standard feeding schedules (Gemma fish feed twice a day and artemia once a day) and dedicated personnel. Animal experiments were approved by the Austrian federal ministry of education, science, and research (BMBWF) under the project license (GZ: 2021–0.611.632).

### 5.2 Locomotion

Locomotor activity was assessed with the open tank test, as previously described [38]. Briefly, zebrafish were placed individually in the center of a large tank (37.5 × 21.5 × 15 cm; length × width × height) covered with foam rubber on the inside of the walls to avoid aggression‐provoking reflections and videotaped for 5 min using a Basler GigE color camera (Noldus, Wageningen, Netherlands). Ethovision XT 12 videotracking software (Noldus) was used to analyze the swimming trajectory of the fish and to calculate total distance moved, time spent in the center zone of the tank, angular velocity, and time spent immobile.

### 5.3 Anxiety‐Like Behavior

Anxiety‐like behavior was measured with the Novel Tank Diving (NTD) test and the Light/Dark Preference (L/D) test, as previously described [38]. For the NTD, fish are placed in a narrow trapezoid tank (19 × 10 × 7 cm; length × width × height) and videotaped from the side for 5 min. Fish that spent more time in the top zone of the tank or that entered the upper part of the tank more frequently were considered less anxious. In the light/dark preference test, the preference of the fish for either side of a custom‐made light–dark arena (45 × 40 × 15 cm; length × width × height) was measured. Fish that spent more time in the light compartment or entered this compartment more frequently were considered less anxious. Ethovision XT 12 (Noldus) was used to analyze the swimming trajectory of the fish during the tests.

### 5.4 Aggressive Behavior

The mirror‐induced aggression paradigm was used to record the fish's interaction with its mirror image and to score the amount of time spent in agonistic behavior [62]. Latency and approaches to the mirror were quantified by Ethovision XT12 videotracking software, while aggressive behavior was evaluated manually by an experienced investigator blinded to the experimental groups.

### 5.5 Social Behavior

To analyze social behavior, we used the corridor social interaction test [21]. In this assay, a test fish is placed in the central compartment of a 3‐chamber clear acrylic tank (50 × 10 × 10 cm; length × width × height) and videotaped for 5 min, followed by Ethovision XT 12 videotracking software (Noldus) analysis. During the first phase of the test (social interaction), one of the two smaller compartments at each end of the tank (10 × 10 × 10 cm; length × width × height) is filled with three unfamiliar stimulus wild‐type fish, while the other compartment remains empty. During the second phase of the test (social novelty) both small compartments are filled with stimulus fish, one with familiar and one with unfamiliar wild‐type fish. Entries into and time spent near the stimulus fish compartments during a 5‐min test period were used as readouts for social behavior.

### 5.6 RNAseq and Bioinformatics

For the RNAseq experiment, zebrafish were euthanised in ice‐cold water (4°C) and brains were extracted in ice‐cold PBS. The telencephalon was micro‐dissected under a stereomicroscope (Bresser stereo microscope science ETD‐201), frozen and stored at −80°C until further processing. RNA from fresh frozen zebrafish brain samples were extracted using peqGOLD TriFast (Life Technologies), followed by DNase digestion (DNA‐free DNA Removal Kit, Life Technologies). High quality RNA samples (RIN 7.4–8.6) were then used for mRNA library preparation and sequenced by paired‐end (2 × 150 bp) sequencing on an Illumina sequencing platform at Novogene Co., Cambridge, UK. The generated paired‐end raw sequence data with 5.30 E+08 total number of reads (mean 6.62E+07 SD 4.38E+06) was quality controlled and sequencing adapters as well as reads shorter than 50 base pairs were removed with Trim Galore! (Galaxy Version 0.6.3.) to increase the mapping quality. We reached on average 33.0 (SD 2.1) million reads over all samples after Trim Galore! On average, 74.9% (SD 1.3%) of the reads could be successfully uniquely mapped with the RNAStar aligner ([63]; Galaxy Version 2.7.8a) to the zebrafish reference genome GRCz11. Final transcript count data was generated with the HTSeq framework ([64]; Galaxy Version 0.9.1) for high throughput sequencing data using standard settings. All analysis were conducted on a private Galaxy instance running on the MedBioNode cluster from the Medical University Graz. Further downstream analysis was conducted with the statistical program R version 4.2.2 within the free RStudio environment. Differential gene expression analysis was performed with DESeq2 (version 1.38.0 [65];) on the count table as output from HTSeq framework. The database for Annotation, Visualization and Integrated Discovery (DAVID; v2024q1 [28]) was used for pathway enrichment analysis by clustering DEGs and associated biological annotation terms into functional groups. Enrichment score cutoff in DAVID was set to 1.3, which corresponds to a corrected *p* value of 0.05. In addition, g:profiler [66] was used for functional enrichment analysis of DEGs.

### 5.7 In Situ Hybridization

In situ hybridization was performed on 14 μm PFA‐fixed frozen brain sections using RNAscope technology (Biotechne, Minneapolis, USA) following the manufacturer's recommendation [67]. Briefly, after pretreatment steps, probe hybridization (RNAscope probe Dr‐lrrtm4l1‐C1) was performed at 40°C in the HybEZ Oven (Biotechne). After several signal amplification steps, sections were visualized using Fast Red (Biotechne) and counterstained using thionine acetate (Merck, Darmstadt, Germany). Specimens were mounted with Entellan mounting medium (Merck) and photographed with a ZEISS Axioscan 7 Microscope Slide Scanner.

### 5.8 Metabolomics

Frozen tissue samples were prepared following the protocol reported by Chetwynd et al. [68], with minor modifications. Briefly, dry weight was recorded for each sample, and tissues were homogenized using Power Bead tubes (Ceramic 2.8 mm, Qiagen) in a mixture of 50% MeOH‐H_2_O on ice. We used a PowerLyzer 24 tissue homogeneiser (Qiagen) and two cycles of 30 s at 3500 rpm. Next, the concentration of each sample was adjusted to 2.5 mg/mL prior to LC–MS acquisition. Targeted analysis of neurotransmitters was performed using a semiquantitative method following previous reports [69]. We used a Waters Cortecs T3 column (100 mm × 3 mm × 2.7 μm, Waters, Manchester, UK), and separation was achieved with a Nexera UHPLC (SHIMADZU, Kyoto, Japan) with a gradient elution using 0.1% formic acid in water and acetonitrile as mobile phases. We used a SCIEX QTRAP 6500 triple quadrupole mass spectrometer (Applied Biosystems, Framingham, MA, USA) operated in positive polarity. Raw files were imported into Skyline software for peak integration, and we used the online tool MetaboAnalyst for further data normalization and statistical analyses. Global unbiased analysis of tissue extracts was performed using a Waters BEH Amide HILIC column (100 mm × 2.1 × 2.1 μM, Manchester, UK) on a Vanquish HPLC coupled to a Q‐Exactive mass spectrometer (ThermoFisher Scientific) operated using a polarity‐switching method. We used a gradient separation with 10 mM ammonium formate +0.1% formic acid and acetonitrile +0.1% formic acid as mobile phases A and B, respectively. The flow was 0.5 mL/min. Ions were generated with HESI and data was collected in the data‐dependent acquisition mode separately for both polarities using the following parameters. In the Full MS settings, the resolution was 140 000, and the AGC target was 3e6, with a maximum IT of 200 ms, in a scan range between 75 and 1050 m/z. The top 3 highest‐intensity precursor ions in a given scan were selected within an isolation window of 2.2 m/z for fragmentation using a stepped collision energy of 20, 40, 100. In the dd‐MS2 settings, the resolution was 17 000, the AGC target was 1e5, and the maximum IT was 50 ms. The intensity threshold to trigger the fragmentation of precursor ions was therefore 1.6e5, and to reduce redundancy, dynamic exclusion lasting 15 s was used. Raw files were processed with mzMine software 4.1.0. Data from both polarities were processed separately. Batching parameters were adapted from Schmid and colleagues [70].

An unbiased MS2 library search was performed for both polarities using Global Natural Products Social Molecular Networking (GNPS), a web‐based application [71], and the publicly available spectral libraries on GNPS. The search options included a precursor ion mass tolerance of 2 Da, fragment ion mass tolerance of 0.5 Da, score threshold of 0.7, and a minimum number of six matched peaks. Spectra were matched against libraries gathered in the *speclibs* collection by GNPS. Libraries running under the names ALL_GNPS, BILELIB19, GNPS‐SCIEX‐LIBRARY, MASSBANK, and MASSBANKEU on GNPS were additionally loaded.

### 5.9 Statistics

Statistical analysis was performed with the Prism 10.2.2 (Systat Software Inc., San Jose, USA) software package. Student's *t* test (two‐tailed) was used to compare data from normally distributed groups, while the Mann–Whitney *U* test was used to compare not‐normally distributed groups. *p*‐values < 0.05 were considered statistically significant.

## Author Contributions

**Eva Tatzl:** conceptualization, investigation, formal analysis, validation, visualization, writing – review and editing. **Giulia Petracco:** investigation, formal analysis, writing – review and editing. **Isabella Faimann:** investigation, formal analysis, writing – review and editing. **Marco Balasso:** investigation, formal analysis, visualization, writing – review and editing. **Agnes Anna Mooslechner:** writing – review and editing. **Thomas Bärnthaler:** visualization, writing – review and editing. **Giovanny Rodriguez‐Blanco:** investigation, formal analysis, resources, visualization, writing – original draft, writing – review and editing. **Florian Reichmann:** conceptualization, funding acquisition, formal analysis, supervision, validation, visualization, writing – original draft, writing – review and editing.

## Conflicts of Interest

The authors declare no conflicts of interest.

## Supporting information


**Table S1.** Telencephalic differentially expressed genes (DEGs) between lrrtm4l1^−/−^ zebrafish and lrrtm4l1^+/+^ zebrafish.

**Table S2.** Peak areas of detected metabolites per sample in the telencephalon of lrrtm4l1^−/−^ zebrafish and lrrtm4l1^+/+^ zebrafish as measured by unbiased metabolomics.

Data S1.

## Data Availability

The data that support the findings of this study are available from the corresponding author upon reasonable request.
