# Supplementary material for: Deficiency of the Synaptic Adhesion Protein Leucine‐Rich Repeat Transmembrane Protein 4 Like 1 Affects Anxiety and Aggression in Zebrafish
Source: Acta Physiol (Oxf). 2025 Apr 4;241(5):e70042. doi: 10.1111/apha.70042 (PMC11970230; doi:10.1111/apha.70042)
Supplement: Supplementary file 3 — Data S1. [file APHA-241-e70042-s003.docx]

**Supplementary information**

Deficiency of the synaptic adhesion protein leucine rich repeat transmembrane protein 4 like 1 affects anxiety and aggression in zebrafish

Eva Tatzl^1^, Giulia Petracco^1^, Isabella Faimann^1^, Marco Balasso^2^, Agnes Anna Mooslechner^1^, Thomas Bärnthaler^1^, Giovanny Rodriguez-Blanco^2^, Florian Reichmann^1,3*^

^1^Division of Pharmacology, Otto Loewi Research Center, Medical University of Graz, Austria

^2^ Clinical Institute of Medical and Chemical Laboratory Diagnostics, Medical University of Graz, Austria

^3^BioTechMed-Graz, Austria

*Corresponding author:

Florian Reichmann, email: florian.reichmann@medunigraz.at


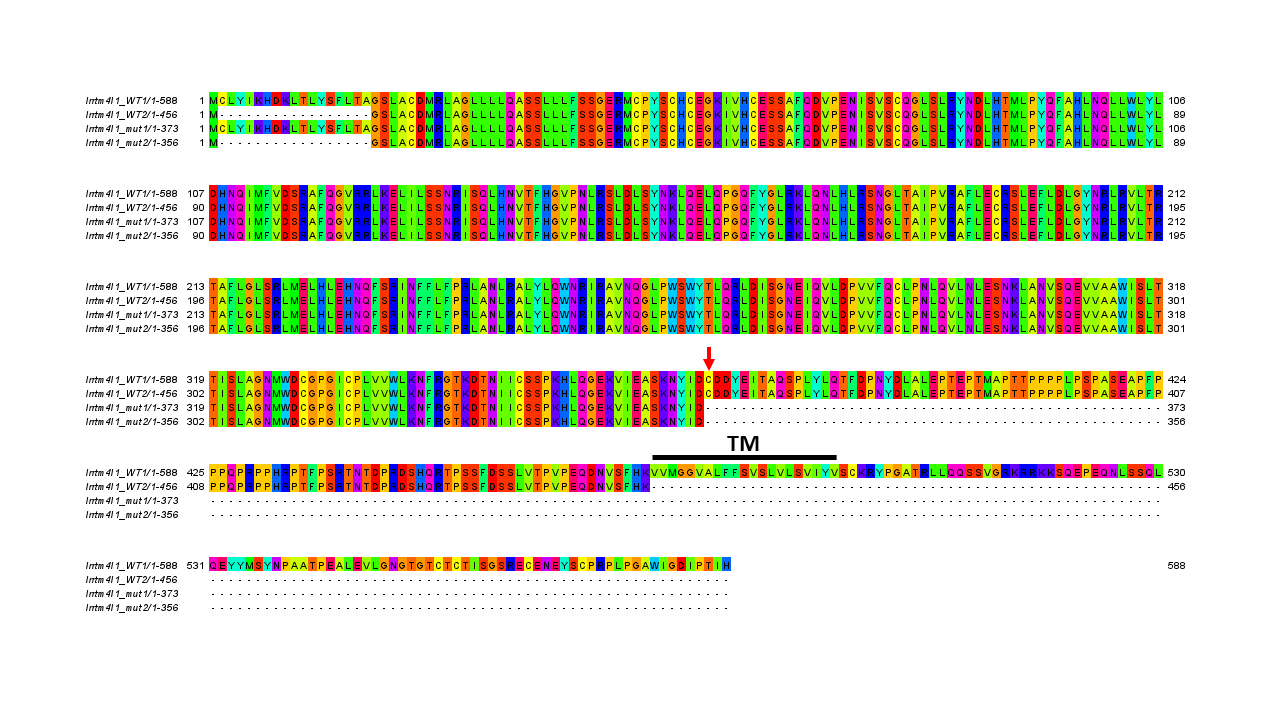


***Supplementary Figure 1. Predicted amino acid sequences of the leucine rich repeat transmembrane neuronal 4 like 1 (lrrtm4l1) mutant alleles.*** *The nonsense mutation in the lrrtm4l1 gene of the zebrafish sa21708 line affects both transcripts of the gene (lrrtm4l1_WT1, ensemble ID: ENSDARP00000101739.4 and lrrtm4l1_WT2, ensemble ID: ENSDARP00000113316.1). Wild-type and mutant (lrrtm4l1_mut1 and lrrtm4l1_mut2) alleles have a conserved amino acid sequence until the premature stop codon indicated by a red arrow. The transmembrane domain (TM) was predicted using DeepTMHMM* (1) *and is highlighted by a black line. The amino acid residues were coloured with the Taylor colour scheme using the open-source Jalview software* (2).


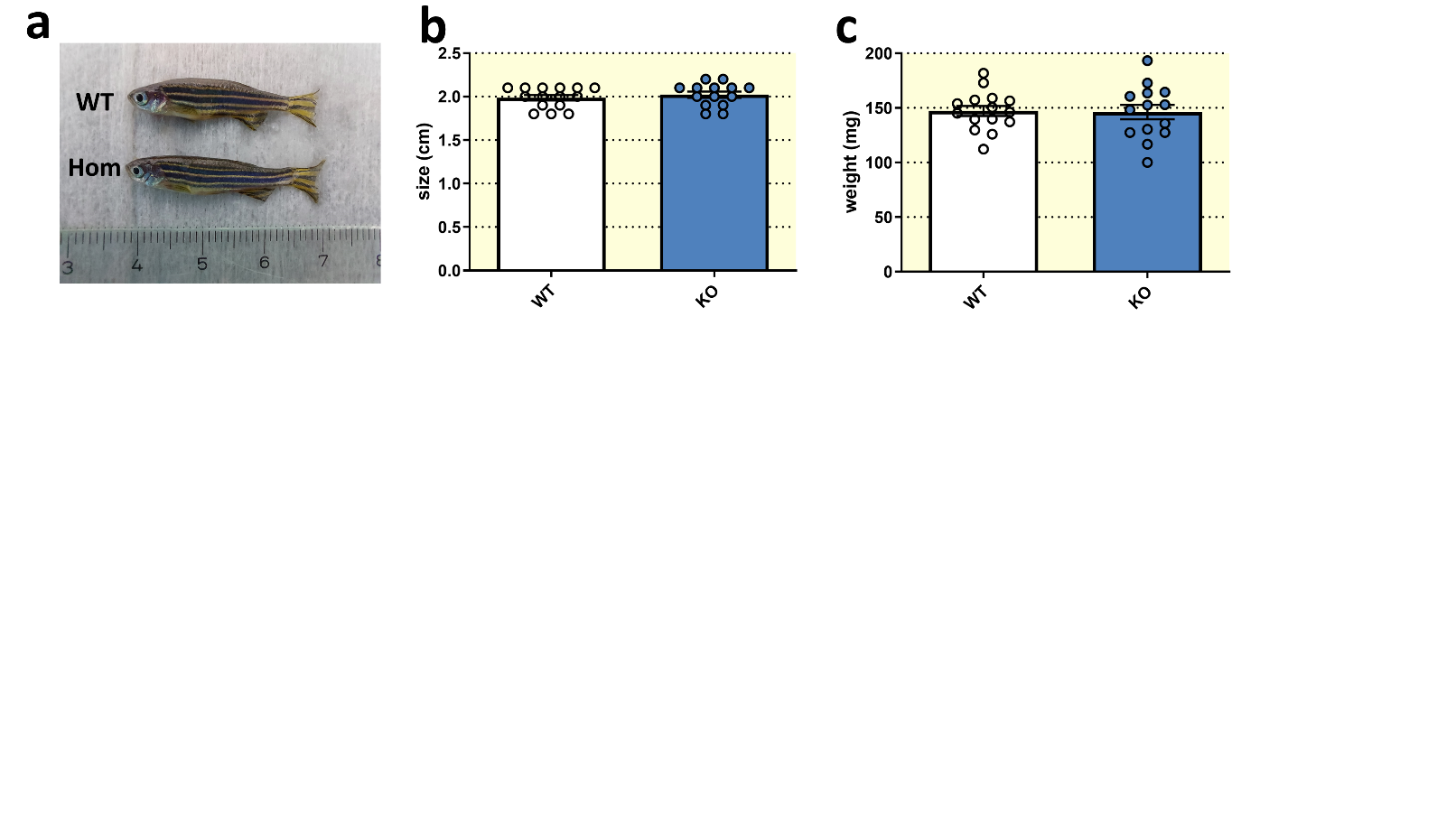


***Supplementary Figure 2. Anatomical features of lrrtm4l1^-/-^ fish.*** *(a) Representative images and comparison between homozygous mutant (Hom) and corresponding wild-type (WT) fish. (b) Standard length and (c) body weight of WT and lrrtm4l1^-/-^ (KO) fish. n=14-15/group. Unpaired t-test. Data are presented as mean ± SEM.*


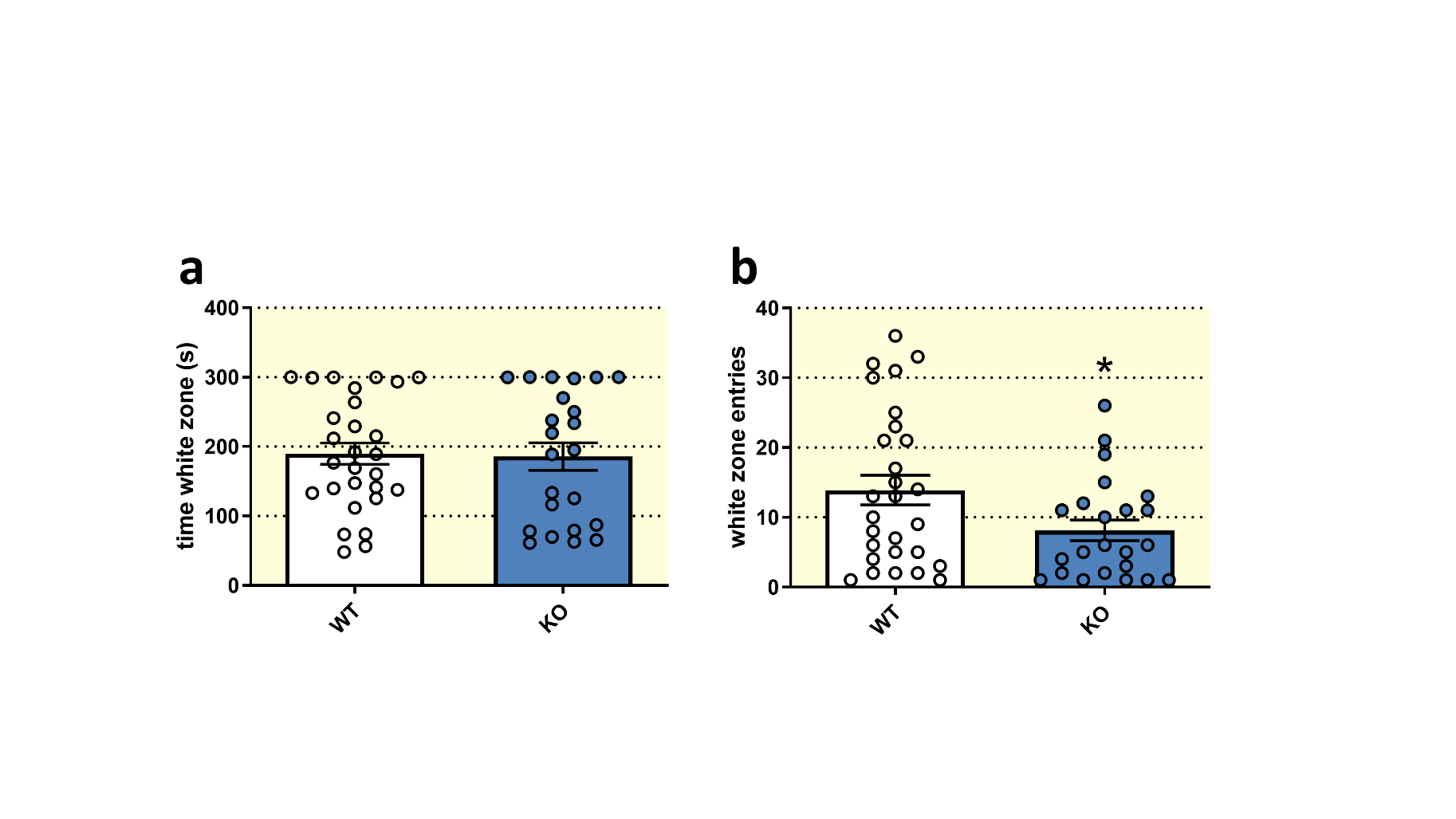


***Supplementary Figure 3. lrrtm4l1^-/-^ zebrafish display signs of heightened anxiety in the light/dark preference (L/D) test.*** *(a) lrrtm4l1^-/-^ (KO) zebrafish spend an equal amount of time in the white zone of the L/D test than lrrtm4l1^+/+^ (WT) zebrafish, but (b) enter the white zone less frequently indicating enhanced anxiety. n = 23-28/group. Student’s t test or Mann-Whitney U test. *P < 0.05 KO vs WT. Data are presented as mean ± SEM*


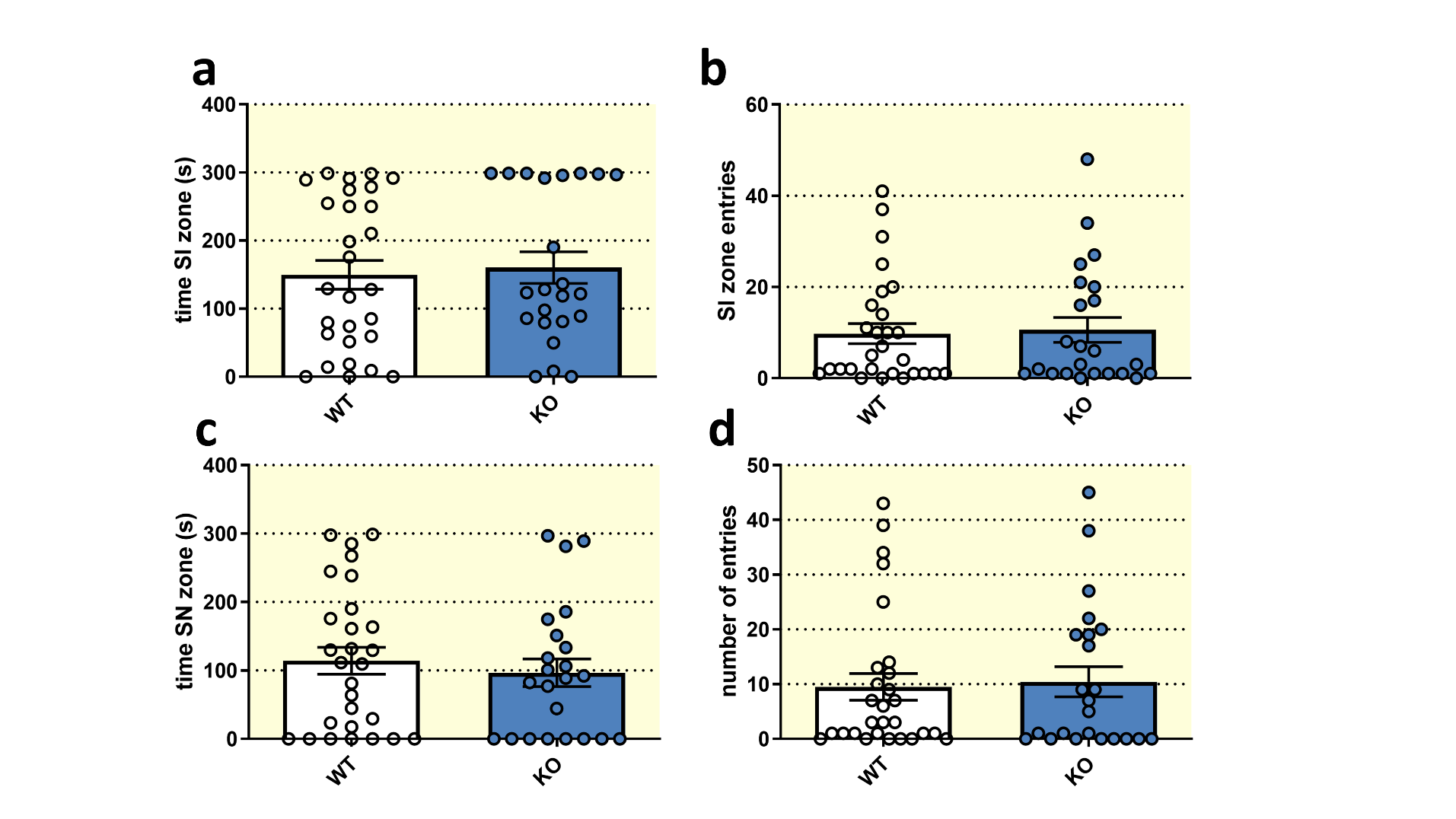


***Supplementary Figure 4. lrrtm4l1^-/-^ zebrafish show no changes in social behaviour during the social novelty assay.*** *(a) Time spent in the social interaction (SI) zone of the test tank. (b) Entries into the SI zone. (c) Time spent in the social novelty (SN) zone of the test tank. (d) Entries into the SN zone. n = 23-28/group. Student’s t test or Mann-Whitney U test. *P < 0.05 KO vs WT. Data are presented as mean ± SEM*


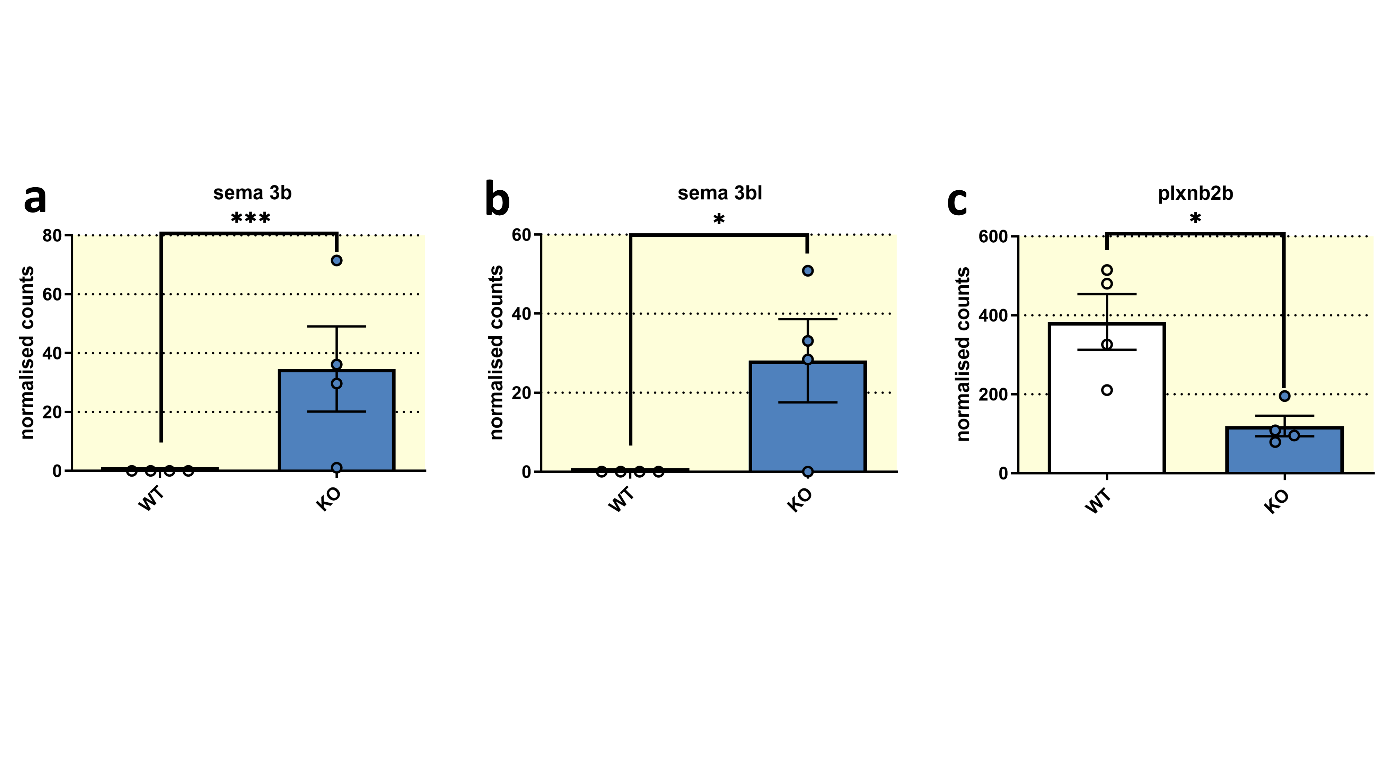


***Supplementary Figure 5. Differentially expressed genes (DEGs) of the semaphorin-plexin pathway.*** *(a) sema domain, immunoglobulin domain (Ig), short basic domain, secreted, (semaphorin) 3B (sema3b) (b) sema domain, immunoglobulin domain (Ig), short basic domain, secreted, (semaphorin) 3bl (sema3bl) (c) plexin b2b (plxnb2b) expression in lrrtm4l1^+/+^ (WT) and lrrtm4l1^-/-^ (KO) zebrafish telencephalon. n = 4/group.* *Wald test with Benjamini-Hochberg correction. ***P<0.001; *P < 0.05 KO vs WT. Data are DESeq2-normalised counts presented as mean ± SEM*


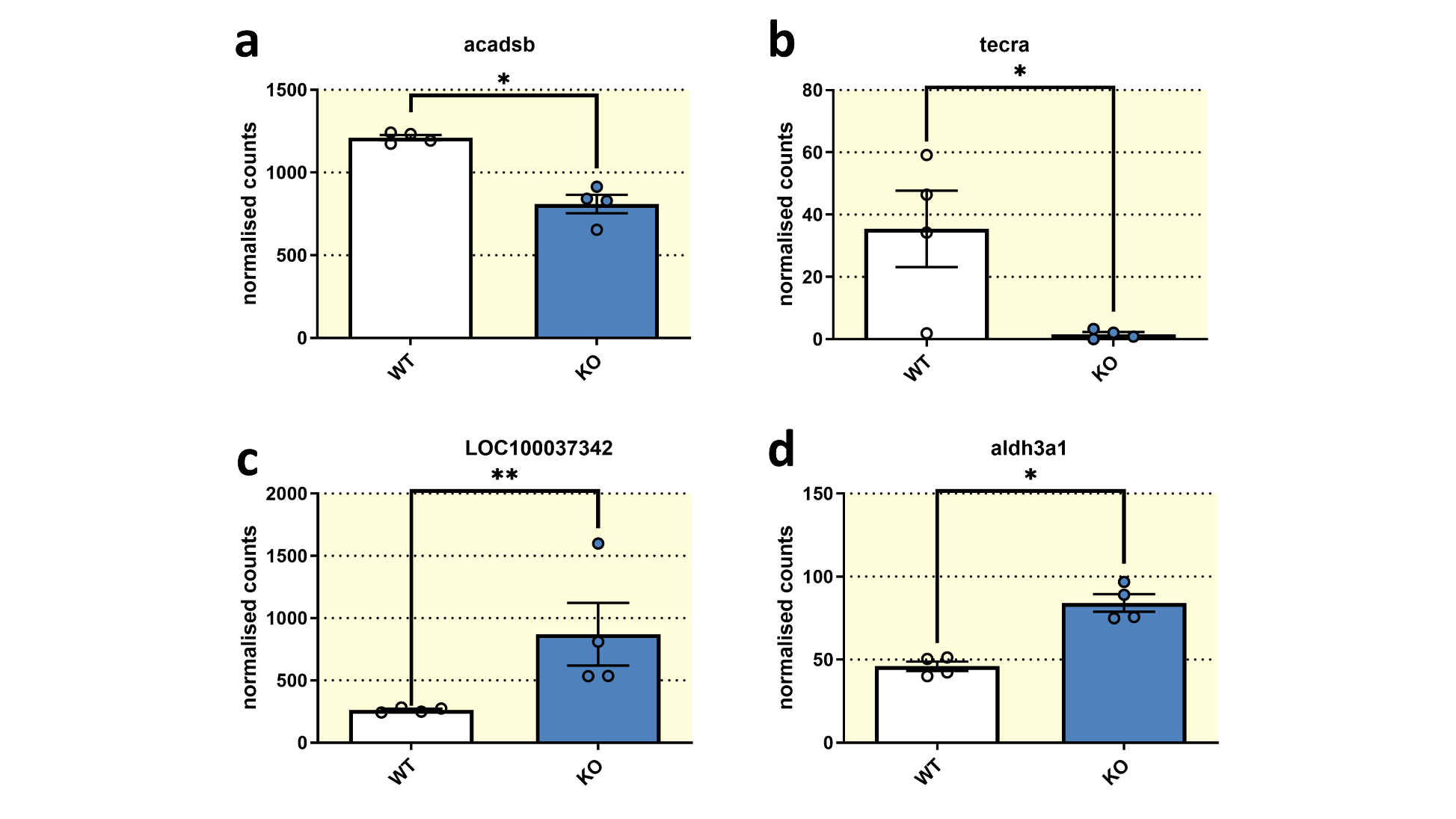


***Supplementary Figure 6. Differentially expressed genes (DEGs) underlying the enriched pathway terms after g:profiler analysis.*** *(a) acyl-CoA dehydrogenase short/branched chain (**acadsb), (b) trans-2,3-enoyl-CoA reductase a (tecra), (c) acyl-CoA dehydrogenase, short/branched chain-like (LOC100037342) and (d) aldehyde dehydrogenase 3 family member A2 (aldh3a1) expression in lrrtm4l1^+/+^ (WT) and lrrtm4l1^-/-^ (KO) zebrafish telencephalon. n = 4/group. Wald test with Benjamini-Hochberg correction. **P<0.01; *P < 0.05 KO vs WT. Data are DESeq2-normalised counts presented as mean ± SEM*


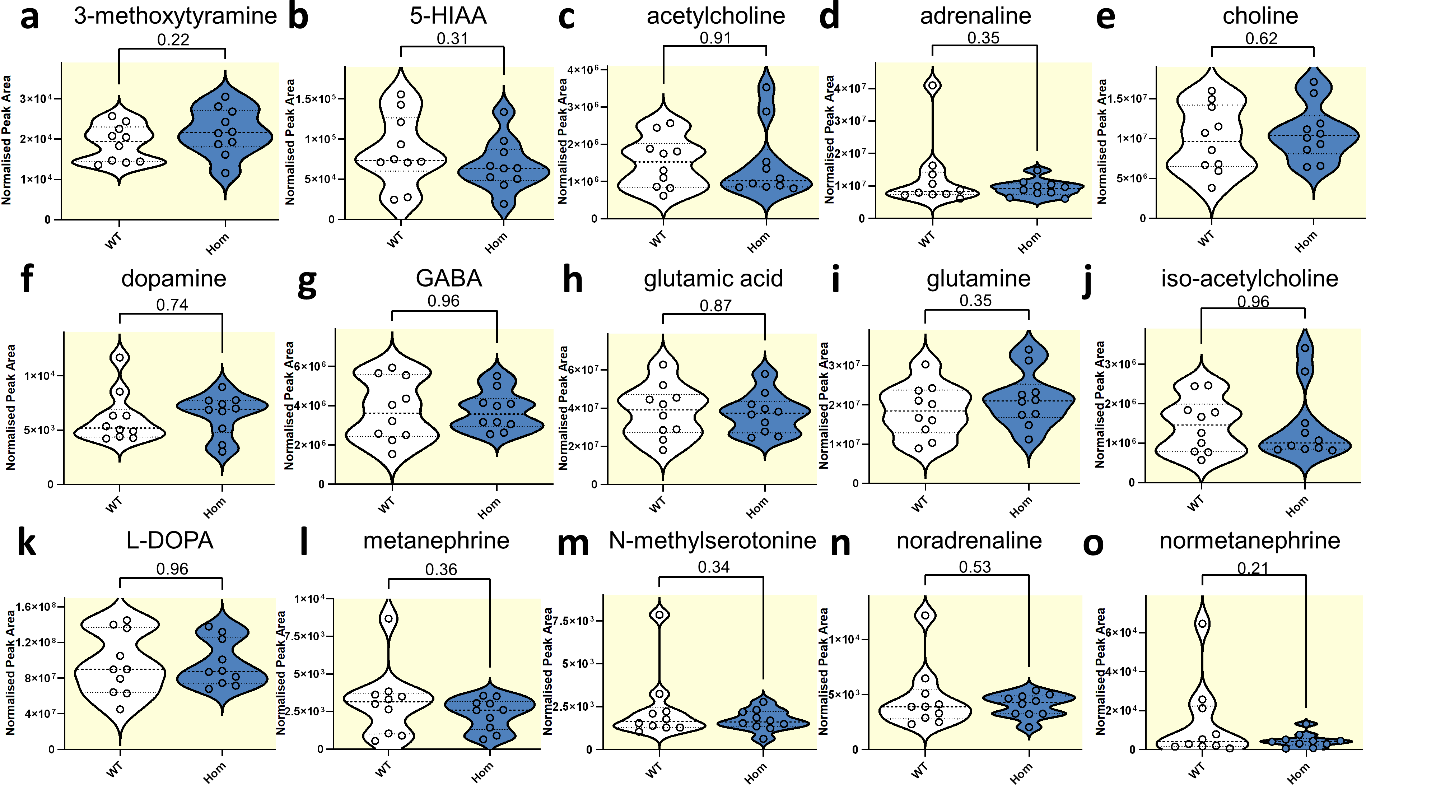


***Supplementary Figure 7. Neurotransmitter-related metabolites in the telencephalon of lrrtm4l1^-/-^ and lrrtm4l1^+/+^ zebrafish.*** *Telencephalic levels of (a) 3-methoxytyramine, (b) 5-Hydroxyindoleacetic acid (5-HIAA), (c) acetylcholine, (d) adrenaline, (e) choline, (f) dopamine, (g) gamma aminobutyric acid (GABA), (h) glutamic acid, (i) glutamine, (j) iso-acetylcholine, (k) l-3,4-dihydroxyphenylalanine (L-DOPA), (l) metanephrine, (m) N-methylserotonine, (n) noradrenaline, (o) normetanephrine as measured by targeted metabolomics in lrrtm4l1^-/-^ (Hom) and lrrtm4l1^+/+^ (WT) zebrafish. n = 10/group. Student’s t test. Data are presented as violin plots with the horizontal black line indicating the median.*

**References:**

(1) Hallgren J, Tsirigos KD, Pedersen MD, Almagro Armenteros JJ, Marcatili P, Nielsen H, et al. DeepTMHMM predicts alpha and beta transmembrane proteins using deep neural networks. bioRxiv 2022:2022.04.08.487609.

(2) Waterhouse AM, Procter JB, Martin DM, Clamp M, Barton GJ. Jalview Version 2--a multiple sequence alignment editor and analysis workbench. Bioinformatics 2009 May 1;25(9):1189–1191.
